# Change in prescription pattern as a potential marker for adverse drug reactions of angiotensin converting enzyme inhibitors

**DOI:** 10.1007/s11096-015-0159-3

**Published:** 2015-07-10

**Authors:** Seyed Hamidreza Mahmoudpour, Folkert W. Asselbergs, Catherine E. de Keyser, Patrick C. Souverein, Albert Hofman, Bruno H. Stricker, Anthonius de Boer, Anke-Hilse Maitland-van der Zee

**Affiliations:** Division of Pharmacoepidemiology and Clinical Pharmacology, Utrecht Institute for Pharmaceutical Sciences (UIPS), Utrecht University, Universiteitsweg 99, David de Wied Building, 3508 TB Utrecht, The Netherlands; Department of Cardiology, Division Heart and Lungs, University Medical Center Utrecht, 3508 GA Utrecht, The Netherlands; Durrer Center for Cardiogenetic Research, ICIN-Netherlands Heart Institute, Utrecht, The Netherlands; Institute of Cardiovascular Science, Faculty of Population Health Sciences, University College London, London, UK; Department of Epidemiology, Erasmus Medical Center, Rotterdam, The Netherlands; Department of Internal Medicine, Erasmus Medical Center, Gravendijkwal 230, Rotterdam, 3015 CE The Netherlands

**Keywords:** ACEI, ADR, Adverse drug reaction, Angiotensin converting enzyme inhibitors, Electronic healthcare database, Positive predictive value, Pharmacoepidemiology, Pharmacovigilance, PPV

## Abstract

*Background* Angiotensin converting enzyme inhibitors (ACEIs) are among the most frequently prescribed groups of medications. ACEI-induced adverse drug reactions (ADRs) are the main reason to discontinue or switch ACEI treatment. ADRs information is not available in prescription databases. *Objective* To identify a proxy for ACEI-induced ADRs in prescription databases. *Setting* The Rotterdam Study is an ongoing prospective cohort study that started in 1990 in the Netherlands and has included 14,926 subjects aged 45 years or older. *Methods* All ACEI starters from 2000 to 2011 were identified using prescription data within the Rotterdam Study. Participants were classified into 4 mutually exclusive groups: continuing, discontinuing, switching to angiotensin receptor blockers (ARBs), and switching to other antihypertensives. For categorization, the maximum time-interval between two prescription periods was set at 3 and 6 months. Subsequently, primary care physician files were searched and clinical events were classified as definite ADRs, probable ADRs, possible ADRs and definite non-ADRs. Finally the accuracy of different prescription patterns as indicators of ADRs was evaluated. *Main outcome measure* Positive predictive values (PPVs), negative predictive values (NPVs), sensitivity and specificity of the prescription patterns of the 4 groups were calculated. *Results* Totally, 1132 ACEI starters were included. The PPV for a definite ADR was 56.1 % for switchers to ARB, while the PPVs for switchers to other antihypertensives, and discontinuation were 39.5 and 19.5 %, respectively. After including probable ADRs and possible ADRs, PPVs for switchers to ARB increased to 68.3 and 90.5 %. A 6-month interval gave slightly higher PPVs compared to a 3-month interval (maximum 6.1 % higher). The differences in NPVs between 3 and 6-months interval groups were approximately 1.0 %. *Conclusions* Switching ACEIs to ARBs is the best marker for ACEI-induced ADRs in prescription databases.

## Impacts on Practice

- Because adverse drug reactions (ADRs) are poorly registered in health care databases, it is difficult to conduct reliable studies of drug-induced ADRs within those databases without suitable proxies.
- In prescription databases, switching from Angiotensin Converting Enzyme Inhibitors to Angiotensin Receptor Blockers is the best indicator for the ACEI-induced adverse drug reactions.
- Applying the validated definition as a marker to investigate genetic and environmental risk factors associated with the occurrence of ACEI-induced ADRs can increase the efficiency of epidemiological and pharmacovigilance studies of ADRs.

## Introduction

Angiotensin converting enzyme inhibitors (ACEIs) are commonly prescribed for a wide range of indications in both cardiovascular and renal disease, including hypertension, heart failure, myocardial infarction, renal failure and diabetic nephropathy [1]. They are first choice in cardiovascular protection in the group of renin angiotensin aldosterone system (RAAS) inhibitors [2]. It has been shown that ACEIs reduce the risk of all-cause mortality and cardiovascular mortality in both patients with hypertension or diabetes mellitus [3, 4].

ACEIs are one of the most frequently prescribed groups of medications worldwide, in the US they were prescribed more than 150 million times per year since 2006 [5]. In the Netherlands there were around 9 million ACEI prescriptions in 2013 [6]. Furthermore, ramipril was the first antihypertensive medication in 2013 with more than 24 million prescriptions dispensed in community pharmacies in the United Kingdom [7]. Adverse drug reactions (ADRs) are one of the main reasons for discontinuation of ACEIs. 19 % of ACEI starters discontinued therapy due to ADRs in a retrospective cohort study of outpatients who were prescribed an ACEI for the first time in a mixed ethnicity US population with 18 months follow-up [8].

Cough is among the most prevalent ADRs to ACEIs with a reported incidence ranging from 5 to 35 %. Cough may occur months and even years after ACEI initiation [9, 10]. More rarely, patients can develop potentially life-threatening angioedema that occurs in an estimated 0.1–0.7 % of patients [11]. Population based studies showed that a large proportion of patients (44.2 %) who discontinued ACEIs switched to an alternative antihypertensive drug within 90 days of discontinuation, indicating that they still need treatment [12]; however reason for discontinuation or switching was not clear in prescription datasets [12, 13]. According to the medical guidelines, ACEIs have to be replaced by Angiotensin Receptor Blockers (ARBs) when ADRs occur [9].

Electronic healthcare and prescription databases have been widely used in ACEIs epidemiologic studies and many of them have been linked to other data including genetic data or laboratory test data [14, 15]. A major difficulty with conducting studies of ADRs is the fact that these are poorly registered in clinical practice, thus health care databases are generally incomplete sources in this respect [16, 17]. Identifying proxies for ADRs based on prescription patterns in prescription databases can facilitate detection of ADRs for pharmacovigilance studies particularly when the dispensing data is linked to other data, like hospital admission data. Such a proxy will also create the opportunity for the large scale studies of biomarkers (such as genetic markers) that might predict the risk of developing ACEI-induced ADRs. ACEI-induced cough can lead to discontinuation of therapy and thereby to a higher risk of cardiovascular events. Angioedema on the other hand is a severe ADR, that might even be life threatening. Other effective antihypertensive drugs are available for patients at risk, and therefore predicting ACEI-induced ADRs is of clinical importance.

### Aim of the study

The objective of this study was to test changes in prescription pattern as an appropriate proxy indicator for detecting the signal of potential ACEI-induced ADRs using data from the Rotterdam Study which contains both detailed drug dispensing data as well as primary care physician data.

### Ethical approval

The Rotterdam Study has been approved by the medical ethics committee according to the Wet Bevolkingsonderzoek: ERGO (Population Study Act: Rotterdam Study), executed by the Ministry of Health, Welfare and Sports of the Netherlands. All participants gave written informed consent to participate in the study, and to obtain information by retrieval of medical records, use of blood and DNA for research purposes, and publication of results, separately.

## Methods

### Data source

The Rotterdam Study is an ongoing prospective cohort study that started in 1990 in Ommoord, a suburb of Rotterdam, the Netherlands. This study has included 14,926 subjects aged 45 years or older. The overall participation was 72.0 % (14,926 of 20,744 eligible invited people). The age distribution and social class of the participants is representative for the Dutch elderly society. The aims and details of the Rotterdam study have been described in detail previously [15, 18]. In the Rotterdam Study, pharmacy dispensing data are available from January 1st, 1991. These records include details about drug names and contents, anatomical therapeutic chemical (ATC)-codes of medications, dosage forms, dispensing dates, number of units dispensed, and prescribed daily dose. Therefore, calculating the duration of drug therapy is possible by dividing the total number of tablets per prescription by the prescribed daily number, so the theoretical end date of prescriptions were calculated accordingly. Additionally the electronic primary care medical records were also available. The electronic medical records contained the notes and diagnoses of the treating primary care physician.

### Study population

A cohort of patients who newly started ACEIs after January 1st, 2000 was identified retrospectively within the Rotterdam Study. The inclusion criteria were: having at least 6 months of valid medication history before starting the ACEI and not having any ACEI prescription within that period to ascertain that they are real ACEI starters. These patients were followed until the end of the study period which was January 1st 2011, or the date a patient died or moved outside of the catchment area (loss of follow-up), whichever came first. Patients whose medical records from general practitioners (GP) were not available were excluded from the study population.

## Outcome measure

**Fig. 1:**
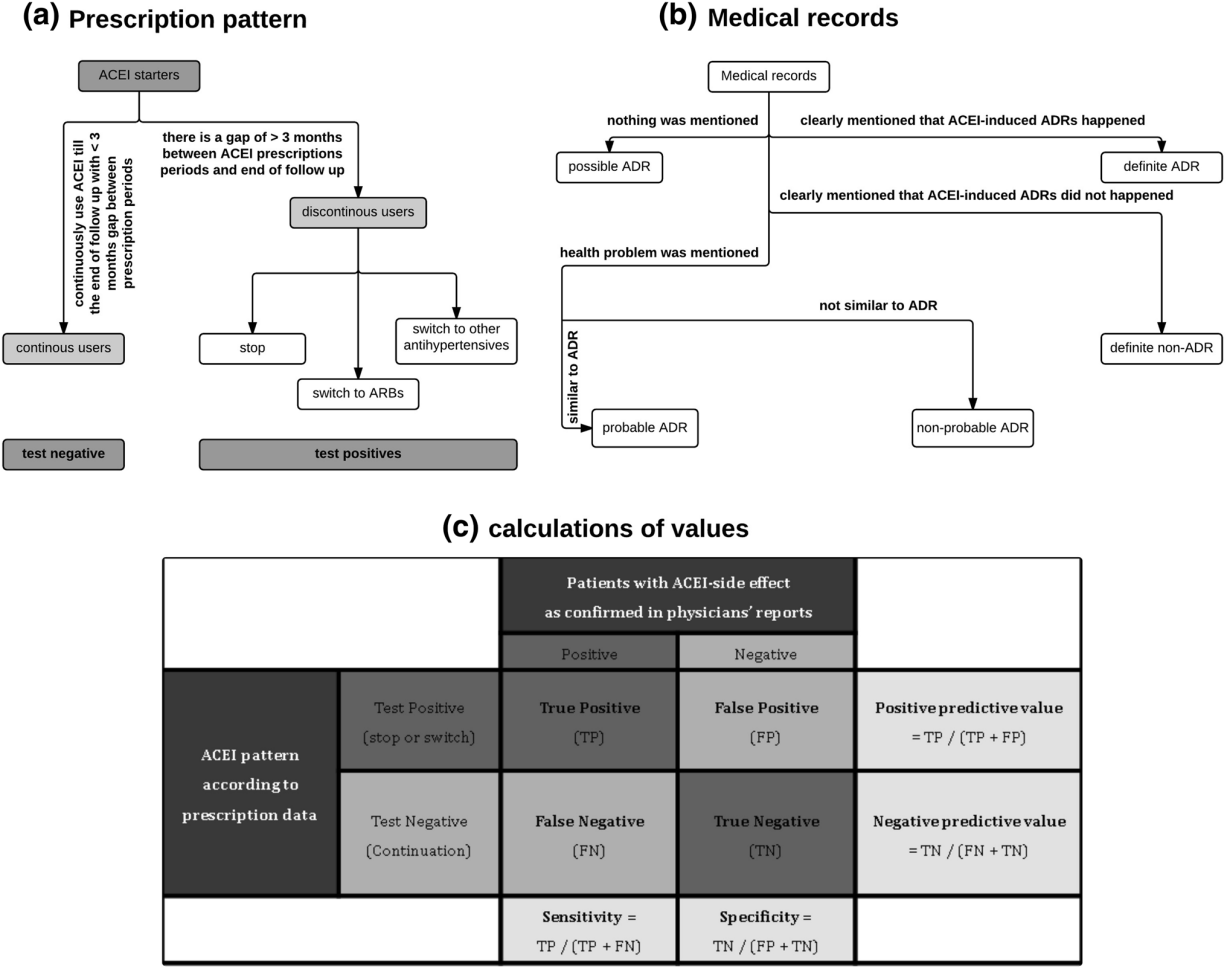
The outcome measurement in both prescription dispensing data and primary care medical records and a table for calculating of the values. *ACEI* angiotensin converting enzyme inhibitors, *ARB* angiotensin receptor blocker, *ADR* adverse drug reaction

Outcomes were measured as below in both, prescription dispensing data and primary care medical records:

A. Prescription dispensing data were identified for all included patients, based on ATC codes including ACEIs (C09A, C09B), ARBs (C09C, C09D), beta blockers (C07), calcium channel blockers (C08), diuretics (C03) and/or antihypertensives (C02). Subsequently the cohort was divided with the following definitions: (Fig. 1a).

  1. Continuation of ACEIs: patients who started ACEIs and continued until the end of the follow up. We allowed a maximum period of 3 months between a renewal of an ACEI dispensing date and the theoretical end date of the previous prescription because the maximum time duration for a prescription to be dispensed in the Netherlands is 3 months. This group was further subdivided into 3 categories depending on their situation when the follow up is ended (end of study, out of study, death). These categories were analysed separately and concomitantly. Furthermore “end of study” and “out of study” groups were analysed together (total minus death), to study whether change in definitions would lead to differences in results. These patients were assumed not to have experienced ADR and they were considered as test negative group.
  2. Discontinuation of ACEIs: patients who did not renew their ACEI prescription within maximum 3 months after the theoretical end date of the last ACEI prescription. Depending on their prescription data within 3 months after the end of ACEIs they were considered as stop (no new antihypertensive), switchers to either ARBs or another antihypertensive drug. These patients were assumed to have possibly experienced an ADR and were considered the test positive groups. The theoretical end date of last ACEI prescription would be the switch date or stop date.
B. In the primary care medical records, for the switching and discontinuation groups, two medical students manually searched 6 month before and 3 months after the switch or stop date to identify the reason for discontinuation or switching of ACEIs. This was done by looking for registered clinical events which might be related to ACEI use. Finally these reports were checked and confirmed by a pharmacist.
  Information from medical records was categorized into 4 groups (Fig. 1b):
  1. Definite ADR: ADR due to ACEI was clearly mentioned in the physician’s records and/or the health problem resolved after discontinuation, thus, the reason for discontinuation was an ADR.
  2. Definite non-ADR: it was clearly mentioned that a physician decided to change or stop medication due to other reasons than an ACEI-induced ADR.
  3. Nothing mentioned: Medical records were available but there was no relevant clinical event mentioned within the required evaluation period. Occurrence of ADR is still possible in this group.
  4. Health problem mentioned: in this category, a clinical event was recorded but it was unclear whether it was due to the use of ACEIs. This category was divided into 2 subgroups according to the characteristics and nature of the mentioned clinical event (probable and non-probable ADR).

## Data analyses

Positive predictive values (PPVs) which are the probability of correctly classifying a patient as having experienced an ACEI-induced ADR were calculated for the test positive groups separately, for these calculations we considered the proportion of test positive cohort (patients discontinued or switched ACEIs) that were identified as definite ADR cases, at least probable ADR cases (definite and probable ADR), and the at least possible ADR cases (definite, probable and possible ADR). Furthermore, PPVs were separately calculated as the proportion of definite ACEI-induced cough cases within patients that discontinued ACEI or switched to other antihypertensives, since this is the most frequently occurring ADR to ACEIs.

In order to calculate sensitivity and specificity, for each patient from the discontinuation or switch group (test positive), a patient from the continuation group (test negative) was selected and medical records were searched from the start date of an ACEI for the same duration of ACEI use that a test positive patient used ACEI; this approach was applied to harmonize the time course between test positive and test negative groups. Sensitivity and specificity were calculated considering definite ADRs only as probable and possible ADRs were not applicable within the continuation group because there was no switch or stop date by definition. Sensitivity in this study was calculated as the proportion of actual ADR cases which are correctly identified as ADR cases and specificity was also calculated as the proportion of non-ADR cases which are correctly identified as non-ADR.

Negative predictive values (NPVs) which are the probability of correctly classifying a patient as not having experienced an ACEI-induced ADR were calculated in test negative group for the at least possible (only the definite ADR cases were deducted from the total number of patients that continued ACEI use), and for at least probable cases (both the definite ADR cases and the probable ADR cases were deducted from the total). Two sided 95 % confidence intervals (CI) were calculated for PPVs, NPVs, sensitivity and specificity (Fig. 1c).

The sensitivity analyses were also performed with a 6 months interval instead of 3 months for defining the prescription patterns.

## Results

### General characteristics and prescription patterns

In total, 1414 ACEI starters were found in the Rotterdam study within the study period; 282 patients (19.9 %) did not have medical records available and finally 1132 patients were included in this study (44.4 % male, mean age 63.7 years). The mean and median follow up time for the included patients were 1602 and 1496 days respectively. Table 1 shows the baseline characteristics and the duration of ACEI use stratified by ACEI use categories. Data are shown both for the 3 and 6 months-time interval between the theoretical end of ACEI prescription and start of a new prescription. For both 3 and 6 months-time intervals, approximately half of the ACEI starters discontinued their medication (55.5 and 48.5 %, respectively) and that the average ACEI treatment duration for all patients was 2 months longer when a 6 months-time interval was applied instead of a 3 months interval. Switchers to ARBs had the shortest mean duration of ACEI use of almost 10 months of ACEI consumption for both the 3- and 6 months interval. When the time interval was changed from 3 to 6 months in the prescription data in total 96 patients changed categories which is 8.5 % of the study population and most of them (82 out of 96) were from the switching or discontinuation group to the continuation group.

**Table 1:** General characteristics of included patients in the study

	Number (% of total)	Mean age (years) [SD]	Gender (% male)	Median ACEI treatment duration (days)	Mean ACEI treatment duration (days) [SD]
*3 Months interval*					
Continuation (N = 503) (44.5 %)					
End of study	267 (23.5)	62 [6.7]	50.2	1219	1340 [1035]
Out of study	135 (12)	62.9 [6.2]	49.6	1350	1451 [1046]
Death	101 (9)	68.8 [7.3]	51.5	508	756 [706]
Stop	308 (27)	64.5 [7]	40.6	207.5	477 [628]
Switch to other antihypertensive than ARB	134 (12)	63.9 [6.5]	45.5	116.5	419 [656]
Switch to ARB	187 (16.5)	62.7 [5.9]	34.2	115	296 [464]
Total	1132 (100)	63.7 [6.9]	44.4	343	785 [909]
*6 Months interval*					
Continuation (N = 585) (51.5 %)					
End of study	299 (26.5)	62 [6.8]	50.5	1207	1347 [1049]
Out of study	167 (14.5)	62.9 [6.1]	48.5	1410	1533 [1047]
Death	119 (10.5)	69 [7.2]	51.3	564	736 [673]
Stop	261 (23 %)	64.3 [6.9]	40.2	179	466 [643]
Switch to other antihypertensive than ARB	106 (9.5)	64 [6.3]	40.6	102.5	343 [532]
Switch to ARB	180 (16)	62.8 [5.7]	34.4	115	293 [473]
Total	1132 (100)	63.7 [6.9]	44.4	397	845 [948]

*ARB* Angiotensin receptor blocker, *SD* standard deviation

### Primary care medical records

Table 2 only shows the detailed categorization of the study population considering the 6 months interval because there were only minor differences between 3 and 6 months interval results. Within the group of definite ADRs, cough and dizziness were the two most prevalent ADRs (73.5 and 4.5 % respectively). Angioedema occurred in 3.0 % of the definite ADRs, and is shown separately as the most dangerous ADR. Details of definite ADRs and probable ADRs are presented in the annotation of Table 2.

**Table 2:** Number of ACEI starters in different categories both according to the prescription data and medical records

Total (N = 1132)	Switchers to ARB (N = 180) (16 %)	Switchers to other antihypertensive than ARB (N = 106) (9.5 %)	Stoppers (N = 261) (23 %)	Continuation (N = 585) (51.5 %)		
				Out of study (N = 167) (14.5 %)	Death (N = 119) (10.5 %)	End of study (N = 299) (26.5 %)
Definite ADR (N = 222) (19.5 %)						
Cough (N = 163)	83	25	35	4	3	13
Angioedema (N = 7)	3	1	1	0	1	1
Others^a^ (N = 52)	15	16	15	3	1	2
Total	101 (56 %)	42 (39.5 %)	51 (19.5 %)	7	5	16
				28 (5 %)		
Definite Non-ADR (N = 48) (4.3 %)						
No need (N = 11)	0	1	10	N/A	N/A	N/A
Not effective (N = 15)	4	4	7	N/A	N/A	N/A
Others^b^ (N = 22)	6	5	11	N/A	N/A	N/A
Total	10 (5.5 %)	10 (9.5 %)	28 (11 %)	N/A		
Nothing mentioned (N = 628) (55.5 %)	40 (22.5 %)	25 (23.5 %)	132 (50.5 %)	124	89	218
				431 (73.5 %)		
Health problem mentioned (N = 234) (20.7 %)						
Probable ADR^c^ (N = 197)	22	20	43	35	21	56
Non-probable ADR^d^ (N = 37)	7	9	7	1	4	9
Total	29 (16 %)	29 (27.5 %)	50 (19 %)	36	25	65
				126 (21.5 %)		

The interval between a renewal of an ACEI prescription and the theoretical end date of the previous prescription was 6 months. *ACEI* Angiotensin converting enzyme inhibitor, *ARB* Angiotensin receptor blocker, *N/A* Not applicable

^a^Allergic reaction (N = 1), Renal dysfunction (N = 2), Runny nose (N = 2), Sexual dysfunction (N = 1), Tiredness (N = 6), Not mentioned (N = 9), Chest pain (N = 1) Decreased taste (N = 1), Dizziness (N = 9), Dizziness plus (N = 1), Gastrointestinal (N = 4), Headache (N = 2), Hiccups (N = 1), Hyperkalemia (N = 2), Itching (N = 3), Itching rash (N = 5), Muscular crumps (N = 1), Nausea (N = 1)

^b^Angioedema history (N = 1), Bad taste (N = 1), Cerebrovascular event (N = 1), Do not like (N = 1), Drug interaction (N = 2), Disease interaction (N = 3), Hypotension (N = 2), Self-stop (N = 3), Surgery (N = 1), Not mentioned (N = 5), Short change (N = 2)

^c^Allergic reaction (N = 1), Angioedema (N = 2), Cough (N = 48), Cough plus (N = 11), Asthma (N = 4), Bronchitis (N = 26), Common cold (N = 6), COPD (N = 8), Dry cough (N = 7), Flu (N = 1), Infectious cough (N = 28), Pneumonia (N = 7), Cough with sputum (N = 4), Dizziness (N = 16), Dizziness plus (N = 1), Dyspnea (N = 5), Hypersensitivity (N = 1), Itching (N = 2), Itching rash (N = 3), Itching throat (N = 2), Rash (N = 3), Tiredness plus (N = 1), Tiredness (N = 3), Sexual dysfunction (N = 2), Shortness breath (N = 5)

^d^Anxiety (N = 1), Bad feeling (N = 6), Body pain (N = 1), Edema (N = 1), Gastrointestinal (N = 5), Hair loss (N = 1), Hospitalization (N = 1), Hand hypoxia (N = 1), Increased blood urea (N = 1), Muscular crumps (N = 5), Muscular pain (N = 1), Nausea (N = 2), Not tolerate (N = 4), Renal dysfunction (N = 3), Runny nose (N = 1), Swollen feet (N = 1), Not mentioned (N = 1) Pulmonary embolism (N = 1)

### Test positive groups

The highest PPVs were found for the switchers to ARBs in all categories [definite ADR 56.1 % (95 % CI 48.8–63.1 %), at least probable ADR 68.9 % (95 % CI 62.0–75.1 %) and at least possible ADR 90.9 % (95 % CI 85.9–94.2 %)]. The PPV for definite ADR was 56.1 % (95 % CI 48.8–63.1 %) when the 6 months-time interval was taken into account which was slightly higher than 55.0 % (95 % CI 47.9–62.0 %) for the 3 months-time interval. Except for the category of at least possible, for all other categories these higher values for the 6 months interval were observed. Cough is the most prevalent ADR of ACEIs, so PPVs for the definite ACEI-induced cough cases were calculated separately. The highest value was 46.1 % (95 % CI 38.9–53.4 %) for the switchers to ARBs considering the 6 months-time interval, in all groups which were considered as test positive, the 6 months interval showed higher PPVs for ACEI-induced cough, (Table 3).

**Table 3:** Positive predictive values for the total adverse drug reactions and cough only cases within the test positive groups (patients who discontinued or switched), Sensitivity and specificity considering the definite adverse drug reactions

	Switchers to ARB	Switchers to other than ARB	Stoppers	Switchers total	Total discontinuation
PPV definite % (95 % CI)					
3 M	55.0 (47.9–62.0)	33.5 (26.1–41.9)	17.5 (13.6–22.1)	46.1 (40.7–51.5)	32.1 (28.5–35.8)
6 M	56.1 (48.8–63.1)	39.6 (30.8–49.1)	19.5 (15.1–24.7)	50.0 (44.2–55.7)	35.4 (31.5–39.5)
PPV at least probable % (95 % CI)					
3 M	68.9 (62.0–75.1)	52.2 (43.8–60.5)	35.7 (30.5–41.2)	61.9 (56.5–67.1)	49.1 (45.2–53.0)
6 M	68.3 (61.2–74.6)	58.4 (48.9–67.4)	36.0 (30.4–42.0)	64.6 (58.9–70.0)	51.0 (46.8–55.1)
PPV at least Possible % (95 % CI)					
3 M	90.9 (85.9–94.2)	83.5 (76.3–88.9)	87.6 (83.5–90.8)	87.8 (83.8–90.9)	87.7 (84.9–90.0)
6 M	90.5 (85.4–94.0)	82.0 (73.7–88.2)	86.5 (81.9–90.2)	87.4 (83.0–90.7)	87.0 (83.9–89.5)
PPV definite Cough cases only % (95 % CI)					
3 M	45.4 (38.4–52.6)	19.4 (13.6–26.9)	11.6 (8.5–15.7)	34.5 (29.5–39.9)	23.3 (20.2–26.8)
6 M	46.1 (38.9–53.4)	23.5 (16.5–32.5)	13.4 (9.8–18.0)	37.7 (32.3–43.5)	26.1 (22.6–29.9)
Sensitivity^a^ definite cases % (95 % CI)					
3 M	91.1 (84.4–95.1)	95.7 (85.7–98.8)	80.6 (69.5–88.3)	92.5 (87.3–95.6)	N/A
6 M	91.8 (85.1–95.9)	93.3 (82.1–97.7)	80.9 (69.5–88.7)	92.2 (86.9–95.5)	N/A
Specificity^a^ definite cases % (95 % CI)					
3 M	67.8 (61.9–73.1)	59.7 (53.1–65.9)	53.7 (49.5–57.8)	64.1 (59.7–68.2)	N/A
6 M	68.4 (62.4–73.8)	61.6 (54.1–68.7)	54.2 (49.6–58.7)	65.7 (61.0–70.1)	N/A

*PPV* positive predictive value, *ARB* angiotensin receptor blocker, *CI* confidence interval, *N/A* not applicable

^a^To calculate the sensitivity and specificity, where it was possible, for each patient from the test positive groups (discontinuation or switch), a patient from the test negative group (continuation) was selected and medical records were searched for the same duration of ACEI use

3 M and 6 M denote time intervals in months between a renewal of an ACEI prescription and the theoretical end date of the previous prescription

Sensitivity was 91.8 % (95 % CI 85.1–95.9 %) and specificity was 68.4 % (95 % CI 62.4–73.8 %) in switchers to ARBs when 6 months interval was applied and both were higher compared with the 3 months gap in the definition (Table 3).

### Test negative groups

The differences in NPVs for both at least probable and at least possible between 3 and 6 months interval group were very small (approximately 1 %) and inconsistent. Within the groups, the differences between the highest and lowest NPVs for subgroups of “end of study”, “out of study”, “death”, “total minus death” and “total continuation” were also small with a maximum of 2.4 % (Table 4).

**Table 4:** Negative predictive values within the test Negative group (patients who continued Angiotensin converting enzyme inhibitor)

	End of study^a^	Out of study^b^	Death^c^	Total minus death	Total continuation
NPV at least possible % (95 % CI)					
3 M	95.5 (92.3–97.4)	95.5 (90.6–97.9)	95.0 (88.9–97.8)	95.5 (93.0–97.1)	95.4 (93.2–96.9)
6 M	94.6 (91.4–96.6)	95.8 (91.6–97.9)	95.8 (90.5–98.1)	95.0 (92.7–96.6)	95.2 (93.1–96.6)
NPV at least probable % (95 % CI)					
3 M	77.9 (72.5–82.4)	75.5 (67.6–82.0)	78.2 (69.2–85.1)	77.1 (72.7–80.9)	77.3 (73.4–80.7)
6 M	75.9 (70.7–80.4)	74.8 (67.7–80.8)	78.1 (69.9–84.9)	75.5 (71.4–79.2)	76.0 (72.4–79.3)

*CI* confidence interval, *NPV* negative predictive value

^a^“End of study” group are patients who continued ACEIs till January 1st 2011

^b^“Out of study” group are patients who continued ACEIs till they went out of the area

^c^“Death” group are patients who continued ACEIs till date of death 3 M and 6 M denote time intervals in months between a renewal of an ACEI prescription and the theoretical end date of the previous prescription

## Discussion

Based on PPV, NPV, sensitivity and specificity, this study showed that switching from an ACEI to an ARB allowing 6 months-time interval between last use of ACEI and start of ARB, is the best marker in the prescription database of the Rotterdam Study for ACEI-induced ADRs. This finding offers the possibility to use prescription databases to identify patients who have experienced ACEI-induced ADRs even in the absence of clinical data or specific ADR registrations. This was also demonstrated for ACEI-induced cough specifically, because switchers from ACEIs to ARBs had the highest PPVs among all groups of ACEI prescription patterns for either definite, probable or possible ADRs and also for the definite ACEI-induced cough cases only. A 6 months interval gave slightly higher PPV compared with a 3 months interval, and both sensitivity and specificity were higher using a 6 months interval.

In all studies that compared discontinuation between different classes of antihypertensive drugs ARBs were used without switching or discontinuation for the longest period followed by ACEI, while the time intervals for defining discontinuation or switch in prescription data were not consistent in all of them [12, 19, 20]. In this study, 3 and 6 months-time intervals were used to find the best interval in terms of indicating ADRs and accuracy to include real stoppers, switchers and continuers because previous studies have shown that time-interval influence the categorization in hypertensive therapy [21]. Out of the total 96 patients who changed categories when the interval changed from 3 to 6 months, 82 changes (85.5 %) were from groups of switching and discontinuation to the continuation group, which suggests that using the 6 month interval is probably better to prevent misclassification because patients who restart are not expected to have stopped due to an ADR previously. Morimoto et al. investigated ACEI-induced ADRs and found that 32.4 % of ACEI starters discontinued ACEI, of whom 19 % discontinued use, due to ADRs after a maximum of 18 months follow up [8]. In our study, 48.5 % of the ACEI starters discontinued their ACEI when the 6 months-time interval was used and ACEIs were on average used for about 28 months in the whole study population when considering a maximum interval of 6 months within the prescriptions.

Other examples where prescription data were validated as a marker for clinical events have been published. For instance, in the Rotterdam Study, using repeated nitrate prescription has been shown to be a suitable marker for angina pectoris in electronic healthcare databases [22] and also changes in prescription data were used previously as an indicator of ADR due to statins [23]. In a sample of 63 cases that switched, discontinued or reduced the dose of their statin therapy, 68 % suffered from ADRs induced by statins and this proxy was used within prescription data for genetic association studies where large numbers of cases are needed [24]. This study tried to identify the reason for discontinuation and switching in general practitioners (GP) files to find the best marker in prescription data for ACEI-induced ADRs, and specifically cough. This study was conducted in the Rotterdam Study, which is a large cohort study within the Netherlands with a good generalizability to the Caucasian population of 45 years and older [25], so the results can be translated to other similar databases.

Pharmacies in the Netherlands are allowed to deliver medication for a maximum of 90 days; therefore the regular time interval for refilling a prescription is 3 months. Results of this study should be used with caution in countries with different intervals for prescription refill. Additionally the proxy cannot differentiate between the different ACEI-induced ADRs, however for cough as the most prevalent ADR, results showed a high predictive value for definite cases [46.1 % (95 % CI 38.9–53.4 %)].

Because usually ADRs are not well registered, the use of electronic healthcare databases can increase the number of cases of ADRs that can be found, and can decrease the amount of time and costs spent in searching for these cases in epidemiologic studies. Many prescription databases can be linked to other types of data, including but not limited to hospital data, genetic data, socio-demographic data and laboratories-test data [26].

Hospital data have been used previously to detect and report ADRs for pharmacovigilance studies [27]. If linkage to hospital data is possible, this can strengthen the validity for the detection of ACEI related ADRs, especially those ADRs that need hospital admission like angioedema. However, for ADRs that do not require hospitalization (like cough) the use of drug dispensing databases might be a good alternative for pharmacovigilance studies.

An important limitation of our study is that only the diagnoses of general practitioners (GP) records were considered. It was not possible to check specialist records or to interview patients. This might have led to misclassification because some clinical events might have been missed, misdiagnosed or not been registered in GP records [28]. The number of general practitioners visited by the patients in the Rotterdam Study is limited to the specific region and that is a single centre study, so the variation in physician’s attitude to diagnose the ACEI-induced ADRs might be less comparing to multicentre studies, however it cannot be ruled out.

## Conclusion

In conclusion, switching from ACEIs to ARBs is the best marker in prescription databases and might be useful to investigate genetic and environmental risk factors associated with the occurrence of ACEI-induced ADRs. Using such data might increase the efficiency of epidemiological studies of ADRs, especially of the ones which are not coded and found back in health care databases.
